# Laparoscopic management of intestinal obstruction in a young adult with a virgin abdomen: Unusual presentation of combined vitellointestinal duct remnants: A clinical case report

**DOI:** 10.1002/ccr3.8395

**Published:** 2024-01-17

**Authors:** Mohannad Al‐Tarakji, Mohamed Almogtaba, Yaseen Al‐Hashimy, Omar S. Moustafa, Mona S. Shehata, Raed M. Al‐Zoubi, Mohamed Said Ghali

**Affiliations:** ^1^ Department of Surgery, Acute Care Surgery Hamad Medical Corporation Doha Qatar; ^2^ Department of Surgery, General Surgery Hamad Medical Corporation Doha Qatar; ^3^ Department of Pharmacy, Woman's Wellness and Research Center Hamad Medical Corporation Doha Qatar; ^4^ Surgical Research Section, Department of Surgery Hamad Medical Corporation Doha Qatar; ^5^ Department of Biomedical Sciences, QU‐Health, College of Health Sciences Qatar University Doha Qatar; ^6^ Department of General Surgery Ain Shams University Cairo Egypt

**Keywords:** abdomen, case report, intestinal obstruction, laparoscopic, management, remnants, vitelline duct

## Abstract

**Key Clinical Message:**

In an 18‐year‐old, Meckel's diverticulum and a rare vitellointestinal fibrous band caused bowel obstruction. Clinicians should be vigilant for such anomalies, especially in young adults with virgin abdomens, as potential sources of intestinal obstruction.

**Abstract:**

In this case report, we highlight the rarity of vitellointestinal or omphalomesenteric duct anomalies causing intestinal obstruction in the adult population. The patient, an 18‐year‐old male, presented to the emergency department with a two‐day history of abdominal pain and vomiting. Physical examination revealed mild distension of his virgin abdomen with generalized tenderness. Abdominal X‐ray displayed dilated small bowel loops, and a computed tomography scan indicated features consistent with closed‐loop bowel obstruction. Diagnostic laparoscopy confirmed a vitellointestinal duct remnant as the cause of the small intestinal obstruction, involving a combined Meckel's diverticulum and vitellointestinal fibrous band. In early fetal development, the vitellointestinal duct communicates between the midgut and the yolk sac, expected to disappear during fetal growth. Failure to obliterate can lead to issues such as intestinal blockage, primarily observed in children, making occurrences in adults, as in this case, infrequent with only a few documented instances. Despite its uncommon occurrence in young adults, healthcare providers should consider the vitellointestinal duct anomalous remnant as a potential source of intestinal obstruction, particularly in individuals with a virgin abdomen. Early detection of intestinal obstruction is imperative for patient survival, facilitating prompt management and minimizing the risk of serious morbidities, ultimately contributing to a better patient outcome.

## Graphical abstract

Unusual laparoscopic management: intestinal obstruction in a young adult.

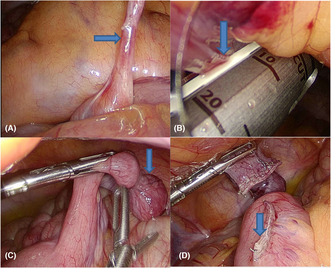

## 1 INTRODUCTION

Intestinal obstruction (IO) is a common surgical emergency that is a major source of morbidity and a costly burden on hospitals worldwide.^1^, ^2^ Adhesions and hernias were found to be the most frequent causes of bowel obstruction.^3^ It was discovered that 2% of infants had vitellointestinal duct (VI), also known as omphalomesenteric duct remnant, which can cause a wide range of symptoms, such as blood in the rectum, abdominal pain, umbilical hernia, and IO. Due to the rarity of VI duct anomalies, these symptoms are infrequent in adulthood; males complain of them more than females do.^4^ VI duct anomalies can be presented in different findings: Meckel's diverticulum, VI fistula, umbilical sinus, umbilical cyst, fibrous cord (complete obliteration of the duct connecting the ileum to the umbilicus), umbilical mucosal polyp. IO due to the VI duct necessitates early diagnosis and proper surgical management or remnant of vitelline vessels.^5^, ^6^ However, instances of intestinal obstruction caused by omphalomesenteric duct anomalies cured with laparoscopic surgery are relatively uncommon. We reported a scarce case of VI duct‐causing closed‐loop intestinal obstruction in an adult patient who presented to our emergency with an acute attack of IO with a long history of abdominal symptoms.

## 2 CASE REPORT

**FIGURE 1:**
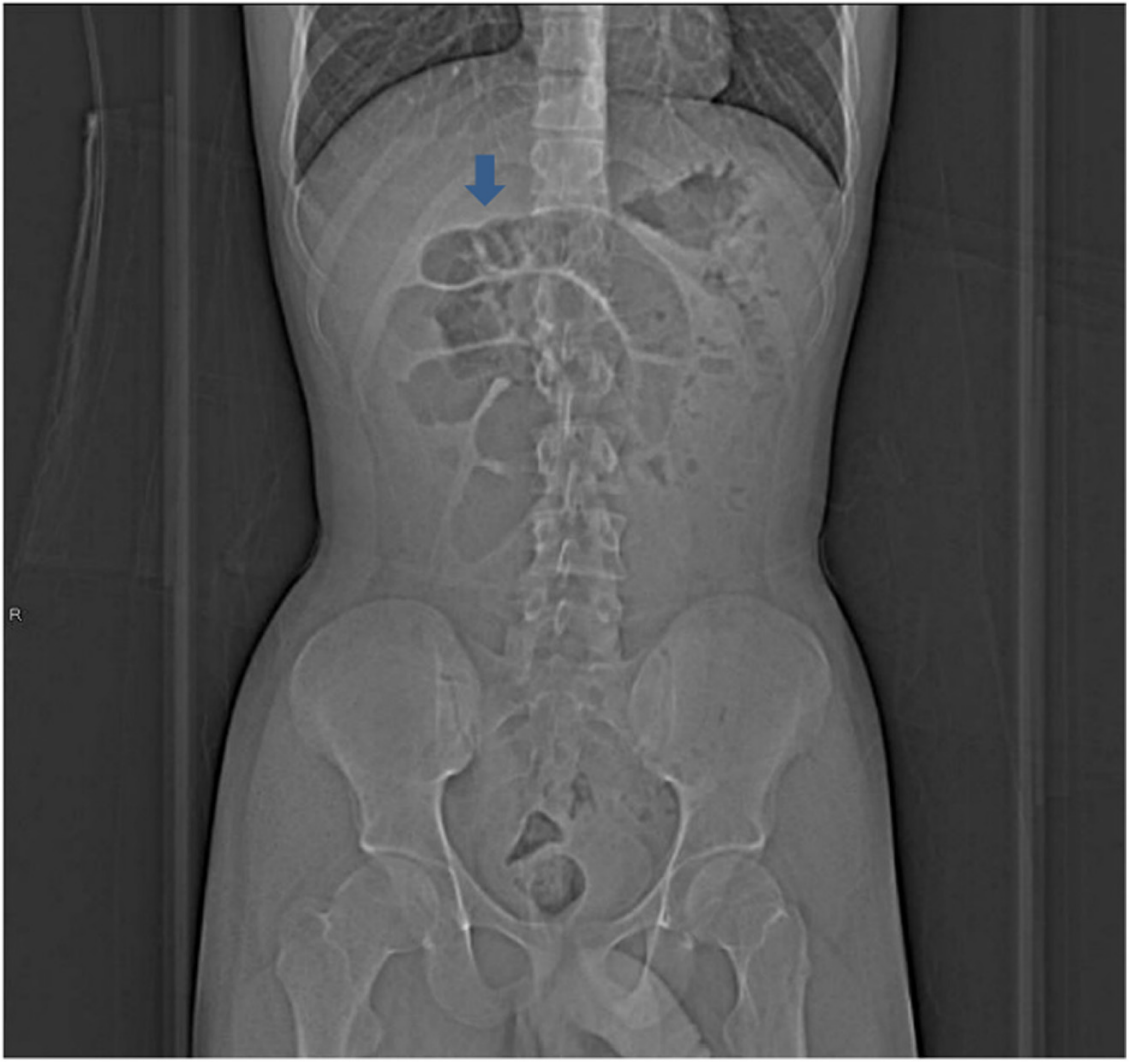
Plan X‐ray abdomen showing dilated intestinal loops as shown by the arrow.

**FIGURE 2:**
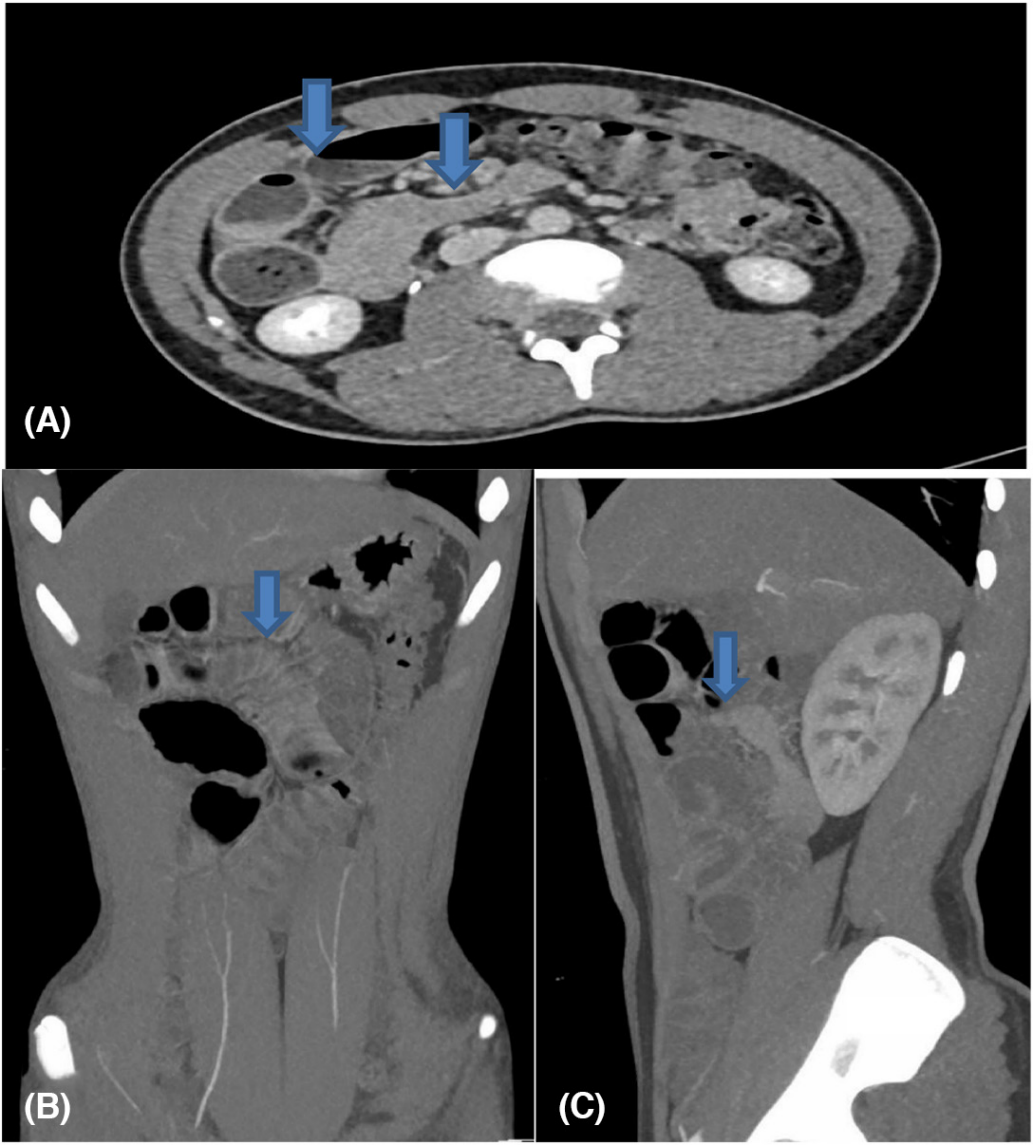
CT Scan abdomen showing findings of intestinal obstruction as mentioned earlier (A) axial view with arrows showed collapsed and distended small bowel (B) coronal view with arrow showing significant dilated jejunal loops and (C) sagittal view showing transitional area between dilated and collapsed small bowel.

An 18‐year‐old male patient with significant intermittent abdomen pain which required repeated hospital visits for the last years, He did not have a clear diagnosis of this pain. He was offered upper and lower GI endoscopy as possible diagnostic tool for his complaints, but the results were negative for any significant pathology. He had an obvious burn scar on his belly were a result of traditional therapy through abdominal skin burns he got in his rural area. He was admitted to our emergency department with complaints of lower abdominal discomfort and vomiting for 2 days (greenish‐yellow vomitus with food particles). He reported passing gas and stool in the morning of admission. He has no past medical or surgical history and has no family history of similar complaints. On admission, the pulse rate was 110 per minute while the rest of the vital signs were within acceptable range. Abdominal examination showed a mildly distended abdomen with generalized tenderness in the lower abdomen with no signs of peritoneal irritation. On auscultation, he had an exaggerated bowel sound. The patient refused a digital rectal examination. Laboratory results showed insignificant numbers; white blood counts (WBCs) were 9.3 × 10^3^/μL, hemoglobin 14.0 gm/d, absolute neutrophil count 7.8 × 10^3^/μL, serum sodium 138 mmol/L, serum potassium 4.1 mmol/L, C reactive protein (CRP) 2.2 mg/L, blood urea 2.5 mmol/L. we asked for an X‐ray abdomen (Figure 1) which showed dilated intestinal loops in the center of the abdomen. CT abdomen with contrast (Figure 2) showed; dilated bowel loops in the right mid and lower abdomen with surrounding minimal fat stranding and a trace of free fluid with a transition point in the mid‐lower abdomen concerning possible closed‐loop obstruction due to herniation of small bowel loops in the right paraduodenal space. After a thorough evaluation of the patient, as well as radiological findings, the decision was made to proceed with surgery, which we advised be done as much as possible via laparoscopy.

## 3 SURGICAL PROCEDURE

**FIGURE 3:**
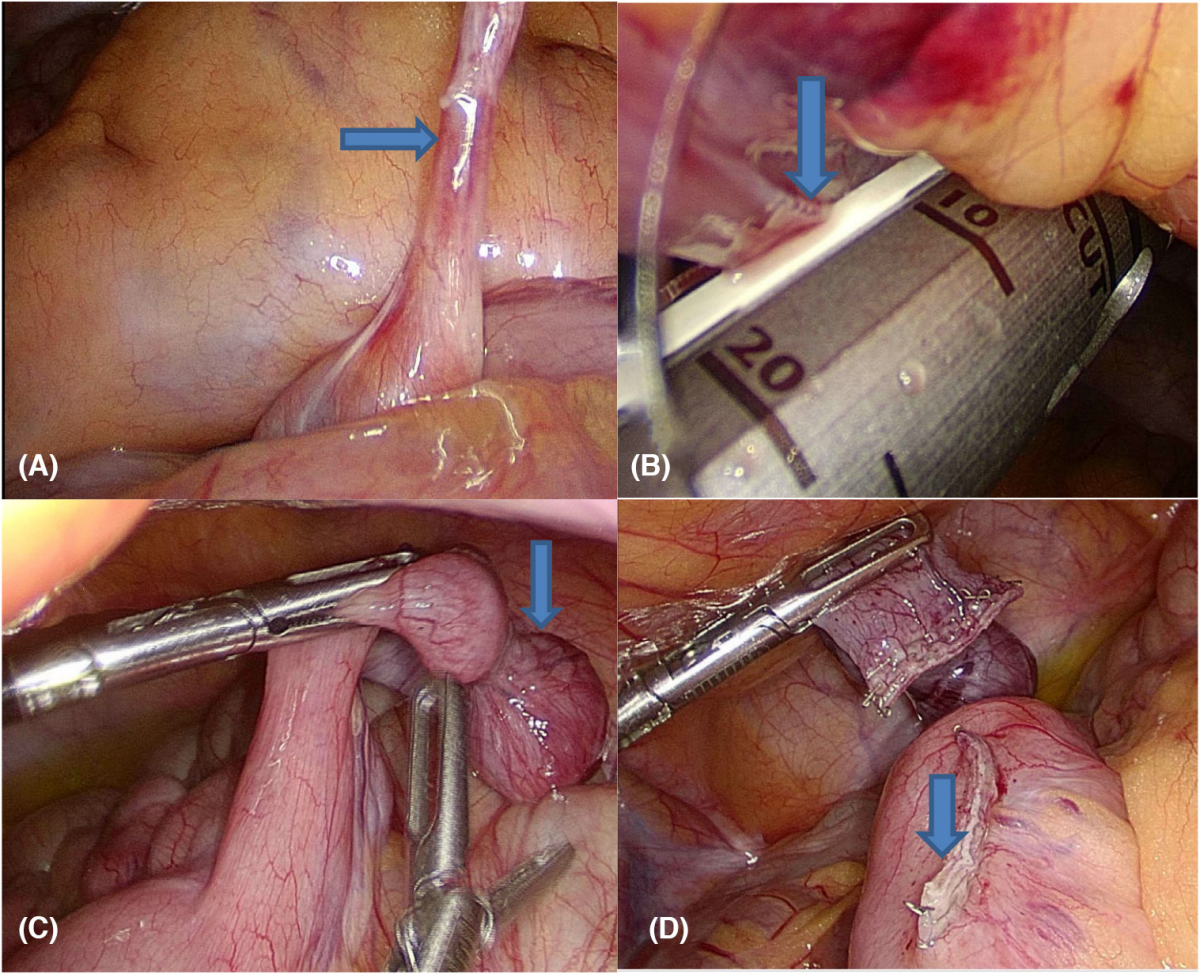
Surgical procedure significant finding and steps (A) Vitellointestinal fibrous band (B) cutting VI band at the umbilical level using stapler (C) Meckel's diverticulum attached to antimesenteric part of small bowel (D) cutting the Meckel's diverticulum using stapler.

The patient was seen by the anesthesiologist, and preanesthesia evaluation was done; American Society of Anesthesiologists (ASA) physical status classification was Class 2E. The patient was in supine position. Through supra‐umbilical transverse skin incision was done,11 mm trocar was introduced by Hasson's open technique. Pneumoperitoneum was created with 14 mmHg abdominal pressure adjustment and insertion of 30‐degree optic camera. We began with diagnostic laparoscopy to confirm the diagnosis and move further if possible: another two 5 mm ports in the left iliac and left lumbar quadrants were inserted under vision. We demonstrated dilated bowel loops, but there was enough room for a full abdominal demonstration. We observed a mixed remnant of the VI duct made up of Meckel's diverticulum and a VI fibrous band‐like structure linking the ileum of about 100 cm proximal to the ileocecal junction to the umbilical area from within, causing small bowel obstruction and proximal dilatation of the small intestine by causing incomplete small bowel volvulus induced by the Meckel diverticulum and the fibrous band. The resection of the Meckel diverticulum and VI duct was done using an (Echelon flex™ endopath) endoscopic Stapler through a lumbar 12 mm port (Figure 3). A thorough bowel and abdominal examination found no further surgical pathology or impairment of the intestine's viability. Following appropriate hemostasis, the port was removed under vision, followed by port site and skin closure. The procedure took 1 h and 25 min and resulted in less than 5 mL of blood loss.

The postoperative course was uncomplicated since the patient tolerated the diet and was able to go home in good condition on the third postoperative day. The specimen had Meckel's diverticulum with gastric heterotopia, according to histopathology. Three weeks after surgery, the patient was reviewed in the clinic, clinically assessed for complete relief of his symptoms, and was permitted to resume normal daily activities.

## 4 DISCUSSION

In this case report, we show an uncommon cause of intestinal Obstruction in an adult patient caused by a combined Meckel's diverticulum and VI fibrous band. In general, IO is a common surgical emergency and a major cause of morbidity and financial expense across the world. Adhesions and hernias are the most common factors leading to IO as reported in the literature.^1^, ^2^, ^3^ Intestinal obstruction, especially the acute form, is induced when the passage of intestinal contents is blocked. The clinical picture of patients with acute intestinal obstruction is commonly colicky abdominal distention, nausea and vomiting, and failure to pass flatus and stool. Patients classically have abdominal distention and tympanic abdomen on percussion and high‐pitched bowel sounds on abdominal examination. In addition to clinical presentation, laboratory investigations include serum lactate level and complete blood count, metabolic panel and correct Imaging is also a factor that can help with the accurate diagnosis and proper management of IO.^7^ Plain radiograph (X‐ray) plays a fundamental role in patients suspected of intestinal obstruction or bowel perforation and directs toward early diagnosis and management. Computed tomography scans are most typically used to confirm the etiology of the blockage and its detrimental consequences, especially the Contrast‐Enhanced Computed Tomography study which gives more details about bowel ischemia and edema.^8^, ^9^, ^10^ In addition to confirmation of the diagnosis, it helps locate the exact site of obstruction and determine if it is complete or partial.^11^ Accordingly, four clinical characteristics are associated with the need for immediate surgical intervention: intraperitoneal free fluid, small bowel feces sign, mesenteric edema, and continuous vomiting.^12^

Embryologically, the VI duct acts as a communicating structure between the yolk Sac and the apex of the midgut loop, and it undergoes obliteration approximately by the seventh week of fetal life. This obliteration process starts from the umbilical site and proceeds until the intestine; therefore, most of the remnants are in the form of Meckel's diverticulum. However, other malformation entities can occur.^13^ Meckel's diverticulum presents 2% of remnant entities of the VI duct that we may face during other emergency surgery as it was considered part of the regular steps of appendectomy to check for its presence, and the symptomatic picture of the VI duct can occur before 4 years of age, while intestinal obstruction presentation is extremely rare in adults.^14^ Presentation of VI duct remnant in adults varies between Abdominal pain, rectal bleeding, umbilical hernia and intestinal obstruction, enteric fistula, Meckel's diverticulum, vitelline cord, umbilical granuloma or umbilical sinus.^4^, ^5^ In this case report, our patient complained of a long history of on‐and‐off abdominal pain that was colicky in nature, particularly with fast food. Such a case with a virgin abdomen and intermittent abdominal pain warrants further investigation to detect the VI pathology and treat it electively rather than waiting for the patient to present in an emergency situation.

Generally, uncomplicated intestinal obstruction management includes intravenous fluids, electrolytes correction, and nasogastric decompression antibiotics coverage of gram‐negative bacteria and anaerobes in case of fever and leukocytosis. However, surgical intervention should be done if the initial management fails or evidence of bowel perforation or hemodynamic instability is noticed especially in the virgin abdomen.^7^

Intestinal obstruction due to the VI duct remnant as a cause can occur by several mechanisms, including; volvulus, intussusceptions, and internal herniation.^5^, ^12^ In our patient, the X‐ray was not conclusive for the diagnosis (Figure 1), and the computed tomography scan showed the possibility of closed‐loop obstruction due to an internal hernia (Figure 2). However, diagnostic laparoscopy confirmed the presence of the mixed remnant of the VI duct made up of Meckel's diverticulum and a VI fibrous band (Figure 3). For the stable patients with nonclear diagnosis, laparoscopic surgery is an effective alternative to laparotomy. The decision to do laparoscopy should be based on the surgeon's experience, as in our case, as well as the available resources at the health facility. In literature, both laparoscopic and open laparotomy are options for the management. Nevertheless, laparoscopic surgery is preferred because of lesser complications. Despite careful attention during laparoscopic surgery is needed, iatrogenic intestinal harm may occur more frequently than with an open procedure due to limited working area and dilated weak intestinal loops.^5^, ^15^

VI duct remnants usually contain heterotopic mucosa that can cause complications in our case there was evidence of gastric mucosa that may lead to ulceration and intestinal bleeding. This is why we prefer complete excision of the VI duct and be sure that no other residual tissue remains not only to be presented by benign nature bleeding but also it was reported before to be presented by malignant neoplasm.^16^, ^17^

In literature, it is documented that VI duct remnant was symptomatic only in 40% of the pediatric group and the rest were discovered incidentally during other operations, but in adult life, it is more in young patients and carries a high risk of morbidity and mortality.^18^, ^19^

We found a few similar case reports in the literature with a similar presentation of intestinal obstruction and a complete diagnosis of a VI duct remnant as a cause, with a few of them managed late with intestinal compromise and bowel resection, and others, like our case, managed early with no required resection and only resection of the VI duct anomalous remnant, but we also reported another finding of VI duct anomalies that it can present as mixture of Meckel's diverticulum and a VI fibrous band.^1^, ^5^, ^14^, ^20^ We believe that our experience in this instance will contribute to the literature by emphasizing the significance of prompt surgical intervention to keep the bowel safe, particularly in patients who complain of long‐term abdominal pain attacks for no apparent cause and not to misinterpret their complaints by nonsignificant symptoms.

## 5 CONCLUSION

Despite its rarity in young adults, the VI duct anomalous remnant should be considered as one of the possible causes of IO, particularly in the virgin abdomen. These cases have presentations that may be misinterpreted as nonspecific abdominal pain by unsuspecting health workers and managed conservatively until it causes major harm to the patient's bowel, so it should be reviewed by a more senior physician to identify it early, manage it without exposing the patients to major morbidities, and ensuring a better patient outcome.

## AUTHOR CONTRIBUTIONS

**Mohannad Al‐Tarakji:** Data curation; formal analysis; investigation; methodology; writing – original draft. **Mohamed Almogtaba:** Data curation; formal analysis; investigation; methodology; writing – original draft. **Yaseen Al‐Hashimy:** Data curation; formal analysis; investigation; methodology; writing – original draft. **Omar S. Moustafa:** Data curation; formal analysis; investigation; methodology; writing – original draft. **Mona S. Shehata:** Data curation; formal analysis; investigation; methodology; writing – original draft. **Raed M. Al‐Zoubi:** Formal analysis; methodology; validation; writing – original draft; writing – review and editing. **Mohamed Said Ghali:** Conceptualization; data curation; formal analysis; investigation; methodology; writing – original draft; writing – review and editing.

## FUNDING INFORMATION

This research did not receive any specific grant from the public, commercial, or not‐for‐profit funding agencies.

## CONFLICT OF INTEREST STATEMENT

The authors of this manuscript have no conflicts of interest to declare. All co‐authors have seen and agree with the manuscript's contents and there is no financial interest to report.

## CONSENT

Written informed consent was obtained from the patient to publish this report in accordance with the journal's patient consent policy.

## Data Availability

Data will be made available on request.
